# Treatment optimization of pelvic external beam radiation and/or vaginal brachytherapy for patients with stage I to II high-risk Endometrioid adenocarcinoma: a retrospective multi-institutional analysis

**DOI:** 10.1186/s12885-021-08524-x

**Published:** 2021-07-04

**Authors:** Wenhui Wang, Lijuan Zou, Tiejun Wang, Zi Liu, Jianli He, Xiaoge Sun, Wei Zhong, Fengju Zhao, Xiaomei Li, Sha Li, Hong Zhu, Zhanshu Ma, Shuai Sun, Meng Jin, Fuquan Zhang, Xiaorong Hou, Lichun Wei, Ke Hu

**Affiliations:** 1 Department of Radiation Oncology, Peking Union Medical College Hospital Chinese Academy of Medical Sciences & Peking Union Medical College, No. 1 Shuaifuyuan Wangfujing Dongcheng District, Beijing, People’s Republic of China; 2 Department of Radiation Oncology, The Second Hospital of Dalian Medical University, Dalian, People’s Republic of China; 3 Department of Radiation Oncology, The Second Hospital Affiliated by Jilin University, Changchun, People’s Republic of China; 4 Department of Radiation Oncology, First Affiliated Hospital of Xi’an Jiaotong University, Xi’an, People’s Republic of China; 5 Department of Radiation Oncology, The General Hospital of Ningxia Medical University, Ningxia, People’s Republic of China; 6 Department of Radiation Oncology, The Affiliated Hospital of Inner Mongolia Medical University, Hohhot, Inner Mongolia People’s Republic of China; 7 Gynaecological Oncology Radiotherapy, The Affiliated Cancer Hospital of Xinjiang Medical University, Urumqi, People’s Republic of China; 8 Department of Radiation Oncology, Gansu Provincial Cancer Hospital, Lanzhou, Gansu People’s Republic of China; 9 Department of Radiation Oncology, Peking University First Hospital, Beijing, People’s Republic of China; 10 Department of Radiation Oncology, The 940th Hospital of Joint Logistics Support force of Chinese People’s Liberation Army, Lanzhou, Gansu People’s Republic of China; 11 Department of Radiation Oncology, Xiangya Hospital Central South University, Changsha, Hunan People’s Republic of China; 12 Department of Radiation Oncology, Affiliated Hospital of Chifeng University, Chifeng, Inner Mongolia People’s Republic of China; 13 Department of Radiation Oncology, The First Affiliated Hospital of Sun Yat-sen University, Guangzhou, Guangdong China; 14 Department of Radiation Oncology, Xijing Hospital, Air Force Medical University of PLA (The Fourth Military Medical University), Xi’an, People’s Republic of China

**Keywords:** Endometrioid adenocarcinoma, High-risk, Stage I to II, Pelvic external beam radiation (EBRT), Vaginal brachytherapy (VBT)

## Abstract

**Background:**

For stage I to II high-risk endometrioid adenocarcinoma patients, the optimal adjuvant radiotherapy modality remains controversial. The present study sought to optimize the treatment of pelvic external beam radiation (EBRT) with/or vaginal brachytherapy (VBT) for high-risk endometrioid adenocarcinoma patients in multiple radiation oncology centers across China.

**Methods:**

This article retrospectively reviewed stage I to II patients with resected endometrioid adenocarcinoma treated at 13 radiation centers from 1999 to 2015. Patients were eligible if they had high-risk features (stage IB Grade 3 disease or stage II Grade 1–3 disease) on the basis of ESMO-ESGO-ESTRO risk group consensus.

**Results:**

A total of 218 patients were included. Fifty-one patients received EBRT, 25 patients received VBT, and 142 patients were administered EBRT combined with VBT. The three groups were comparable in baseline characteristics, except the proportion of stage IB and Grade 3 disease in the VBT group was significantly higher and their age was older. Survival analysis showed that OS, DFS, LRFS and DMFS were significantly different among the three groups. Two out of three groups were compared with each other, and results demonstrated that DFS, LRFS and DMFS were worse in the VBT group than in the EBRT or EBRT + VBT group. The 3-year OS rates were 95.2, 85.2 and 95.1% in the EBRT, VBT and EBRT + VBT groups, respectively (*p* = 0.043). There was no significant difference in survival outcomes between EBRT group and EBRT + VBT group. A propensity matching analysis was performed to eliminate group differences. The results demonstrated that DFS and LRFS were significantly improved in the pelvic radiation group compared to the VBT group. Distant failure accounted for most of the failure patterns. Patients in the VBT group had significantly increased local and regional recurrence rates than patients in the EBRT or EBRT + VBT group. Acute and chronic radiation-induced toxicities were well tolerated for all patients.

**Conclusion:**

For patients with postoperative stage I to II high-risk endometrioid adenocarcinoma, compared with VBT alone, radiotherapy modalities including EBRT significantly improved DFS, LRFS and DMFS with tolerable adverse effects. Overall survival was not significantly different between EBRT and EBRT + VBT modalities.

## Background

Endometrial cancer (EC) is a common gynecological cancer across China, among which endometrioid adenocarcinoma accounts for most [1]. Surgery is the radical curative method. Adjuvant radiotherapy is recommended for specific patient subgroups. The radiation modality consists of pelvic external beam radiotherapy (EBRT) to the pelvis and vaginal brachytherapy (VBT) to the vaginal cuff to clear up microscopic disease in the locoregional area.

The choice of adjuvant radiotherapy modality is risk-based. In 2016, the European Society for Medical Oncology, European Society for Radiotherapy & Oncology and European Society of Gynaecological Oncology (ESMO-ESGO-ESTRO) consensus was devised and classified endometrial cancer into four risk groups according to FIGO stage, depth of myometrial invasion, differentiation grade, tumor type and lymphovascular invasion (LVSI) [2]. Patients with low-risk endometrial cancer have a low recurrence rate and are managed by surgery alone [3]. For patients with intermediate- to high-risk factors, several trials [4–6] have compared treatment with EBRT to no further therapy and concluded that EBRT significantly reduced the local-regional recurrence rate among these patients. For patients with intermediate or high-intermediate risk factors, prospective randomized studies of slightly different patient populations [7–9] have demonstrated that VBT provides a comparable reduction in vaginal recurrence to pelvic radiation and that pelvic recurrence rates are low among patients treated with vaginal cuff brachytherapy. VBT is recommended for this patient risk group.

High-risk endometrial cancer is characterized by an increased risk of pelvic recurrence and distant metastases that contribute to the inferior outcomes of this group. However, due to the small number of high-risk patients in previous prospective studies, the optimal mode of radiotherapy for this group of patients remains controversial.

The present study aimed to compare the oncologic outcomes of EBRT, EBRT + VBT and VBT alone for high-risk patients with stage I to II endometrioid adenocarcinoma treated at multiple radiation oncology centers across China.

## Methods

### Patient selection and eligibility criteria

**Fig. 1:**
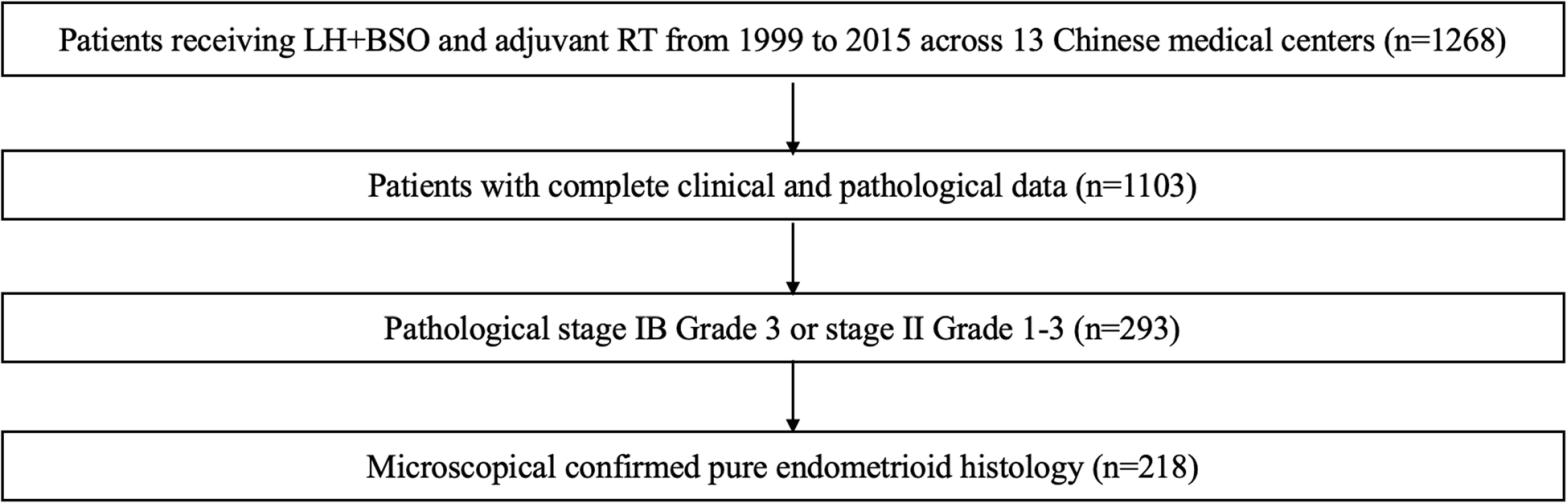
Cohort identification algorithm

This was a retrospective multicenter study with 13 participating Chinese radiation oncology centers of grade A tertiary hospitals. The clinical trial ID of the study is ChiCTR-PRC-17010712. Between Jan. 1999 and Dec. 2015, all patients with stage I to II endometrioid adenocarcinoma after surgery and postoperative radiotherapy were analyzed. Patients were eligible if they had been stratified into a high-risk group on the basis of ESMO-ESGO-ESTRO risk group consensus. High-risk features in our research were (1) stage 1B Grade 3 disease or (2) stage II disease. Patients with the following clinical scenarios were excluded: serous, clear cell carcinoma or carcinosarcoma; a history of a previous malignancy; previous radiotherapy, hormonal or chemotherapy treatment; palliative resection; missing data (Fig. 1). Patients were restaged based on criteria from the 2009 International Federation of Gynecology and Obstetrics (FIGO) for uterine carcinomas. This study was approved by the Institutional Review Board of Peking Union Medical College Hospital (N0. S-K139).

### Treatment approaches and follow-up

All patients underwent total hysterectomy with bilateral salpingo-oophorectomy (TH+ BSO). Lymphadenectomy or suspicious lymph node biopsy was performed as a routine procedure when positive preoperative and intraoperative findings existed. For patients without lymph node dissection or sampling, pelvic and abdominal MRI, CT or PET-CT was required to confirm that there was no lymph node metastasis pre- or post-surgery.

Radiotherapy was administered to all patients. Pelvic external beam radiation with/or vaginal brachytherapy was performed according to the patients’ pathological characteristics, physical status, willingness and the doctors’ preference. The clinical target volume (CTV) consists of the upper part of the vaginal stump and regional lymphatic drainage regions, including the common iliac, internal iliac, external iliac, obturator, and presacral areas. Treatment planning was performed by the CT-based intensity-modulated radiotherapy (IMRT) technique, three-dimensional conformal radiotherapy (3D-CRT) modality or a four-field box technique. High-dose rate (HDR) brachytherapy was delivered with a vaginal cylinder to the upper part of the vagina. The indication of chemotherapy was based on the doctor’s recommendations, pathology findings, intraoperative conditions, the patient’s physical condition and willingness. Intravenous concurrent or sequential adjuvant chemotherapy consisted of carboplatin/paclitaxel, cisplatin/doxorubicin, cisplatin/doxorubicin/paclitaxel and other platinum based regimens. Therapy related toxicities were evaluated by the Common Terminology Criteria for Adverse Events Version 4.0 (CTCAE 4.0).

Patients follow up every 3–6 months for 2 years, then every 6 months for 3 years, then annually. Examinations for follow-up included: gynecological physical examination, pelvic and abdominal ultrasound or CT and chest X-ray or CT. MRI or PET-CT was performed as clinically indicated. Laboratory tests included: blood routine, hepatic and renal function and tumor makers, including CA125, et al.

### Data analysis

Survival durations were defined as the time from surgery to the date of death due to any cause or last follow-up time (overall survival, OS), to the date of treatment failure or death due to any cause or last follow-up time (disease-free survival, DFS). Local-regional failure-free survival (LRFS) was calculated from the date of surgery to the date of vaginal stump recurrence or regional lymphatic drainage area failure or death due to any cause or the last follow-up time. Distant metastasis failure-free survival (DMFS) was calculated from the date of surgery to the date of distant metastasis failure or death due to any cause or the last follow-up time.

Data analysis was performed by SPSS statistical software (version 25.0; SPSS Inc., Chicago, IL). The chi-square test was used for categorical variables between treatment groups. Analysis of variance (ANOVA) was used to compare normally distributed multigroup variables, and the LSD test was used to compare intergroup variables. LSD value < 0.05 was considered statistically significant. Propensity-matched analysis (PSM) was used to eliminate group differences. Patients in the VBT group were matched one-to-one to patients in the EBRT or EBRT + VBT group based on stage, grade and age at a matching tolerance of 0.02. The Kaplan-Meier method was performed to calculate the survival data, and differences between groups were determined by the log-rank test. A *p*-value of < 0.05 was considered statistically significant.

## Results

### Patients and tumor characteristics

A total of 218 patients with high-risk endometrioid adenocarcinoma were enrolled. The median age was 55 years old (range, 23 to 85 years old). Most patients were in FIGO stage II (140/218, 64.2%), with stage IB accounting for 35.8% (78/218). All patients underwent total hysterectomy and bilateral salpingo-oophorectomy. Lymphadenectomy was achieved in 87.2% (190/218) of all patients. Adjuvant chemotherapy was performed in 42.9% (91/218) of patients.

A total of 51 patients received EBRT (EBRT group), 25 patients received VBT (VBT group), and 142 patients were administered EBRT combined with VBT (EBRT + VBT group). Table 1 summarizes the patients’ characteristics. As to pelvic external radiation dose-fractionation schedule, for EBRT alone group, the median dose of EBRT was 50.0Gy (range, 44.0–54.0Gy), and the median fractions were 25fx (range, 22 – 28fx). For combined EBRT with VBT group, the median dose of EBRT was 50.0Gy (range, 39.6–50.4Gy) while the median fractions were 25fx (range, 22 – 28fx). For VBT as a boost, there were 14 different dose- fractionation schedules while for VBT alone, there were 4 dose-fractionation schedules. The most common prescription schedule for VBT as a boost after EBRT was 5 Gy in 2 fractions (47/142, 33.1%) followed by 5 Gy in 4 fractions (33/142, 23.2%). The most common fractionation for VBT alone was 5 Gy in 6 fractions, accounting for 84.0% (21/25) of all patients followed by 5 Gy in 8 fractions 8.0% (2/25). All practitioners specified dose to 0.5-cm depth from the vaginal surface. Among patients treated with EBRT (193 cases), 95 patients (49.2%) received IMRT, 54 patients (28.0%) received 3D-CRT, and 44 patients (22.8%) received conventional radiotherapy. The proportions of Grade 3 and stage IB disease were significantly higher in the VBT group than in the EBRT or EBRT + VBT groups (*p* < 0.05). In terms of age, patients in the VBT group were older than patients in the other groups. Other baseline characteristics between the three groups were comparable (*p* > 0.05).

**Table 1:** Baseline clinical characteristics for patients treated with adjuvant EBRT with/or VBT

	Patients (***N*** = 218)						
	**EBRT(*n*** **= 51)**		**VBT(*n*** **= 25)**		**EBRT + VBT (*n*** **= 142)**		***p*-value**
**Clinical Characteristic**	No.	%	No.	%	No.	%	
**Age, years**							0.002
Mean	53.31		60.96		54.36		
Range	23–84		42–85		34–75		
**Lymphadenectomy**							0.867
No	7	13.7	4	16.0	17	12.0	
Yes	44	86.3	21	84.0	125	88.0	
Mean	18.69		21.40		18.73		0.054
Range	0–50		0–45		0–65		
**Stage (FIGO 2009)**							0.000
IB	14	27.5	20	80.0	44	31.0	
II	37	72.5	5	20.0	98	69.0	
**Grade**							0.003
1	4	7.8	3	12.0	32	25.5	
2	20	39.2	2	8.0	47	33.1	
3	27	52.9	20	80.0	63	44.4	
**Myometrial invasion**							0.294
< 1/2	13	25.5	4	16.0	43	30.3	
≥ 1/2	35	68.6	21	84.0	95	66.9	
Missing	3	5.9			4	2.8	
**Invasion of lower uterine segment**							0.205
N0	28	54.9	19	76.0	86	60.6	
Yes	23	45.1	6	24.0	56	39.4	
**LVSI**							0.224
No	40	78.4	16	64.0	113	79.6	
Yes	11	21.6	9	36.0	29	20.4	
**Chemotherapy**							0.105
No	26	51.0	19	76.0	76	53.5	
Yes	25	49.0	6	24.0	60	42.3	
Missing					6	4.2	

*Abbreviation*: *FIGO* International Federation of Gynecology and Obstetrics, *EBRT* External Beam Radiation, *VBT* Vaginal Brachytherapy, *LVSI* Lymphovascular Space Invasion

### Effects of EBRT and/or VBT on survival

For all patients, the median follow-up time was 42 months (range, 3–237 months). A total of 16 patients died during follow-up: 2 in the EBRT group, 5 in the VBT group and 9 in the EBRT + VBT group. Secondary cancer appeared in 4 patients. The cause of death was endometrial carcinoma in 14 patients (87.5%), and cardiovascular disease in 2 patients (12.5%). The median OS was not reached, and the 1-, 3- and 5-year OS rates were 99.5, 93.4 and 89.2%, respectively.

**Fig. 2:**
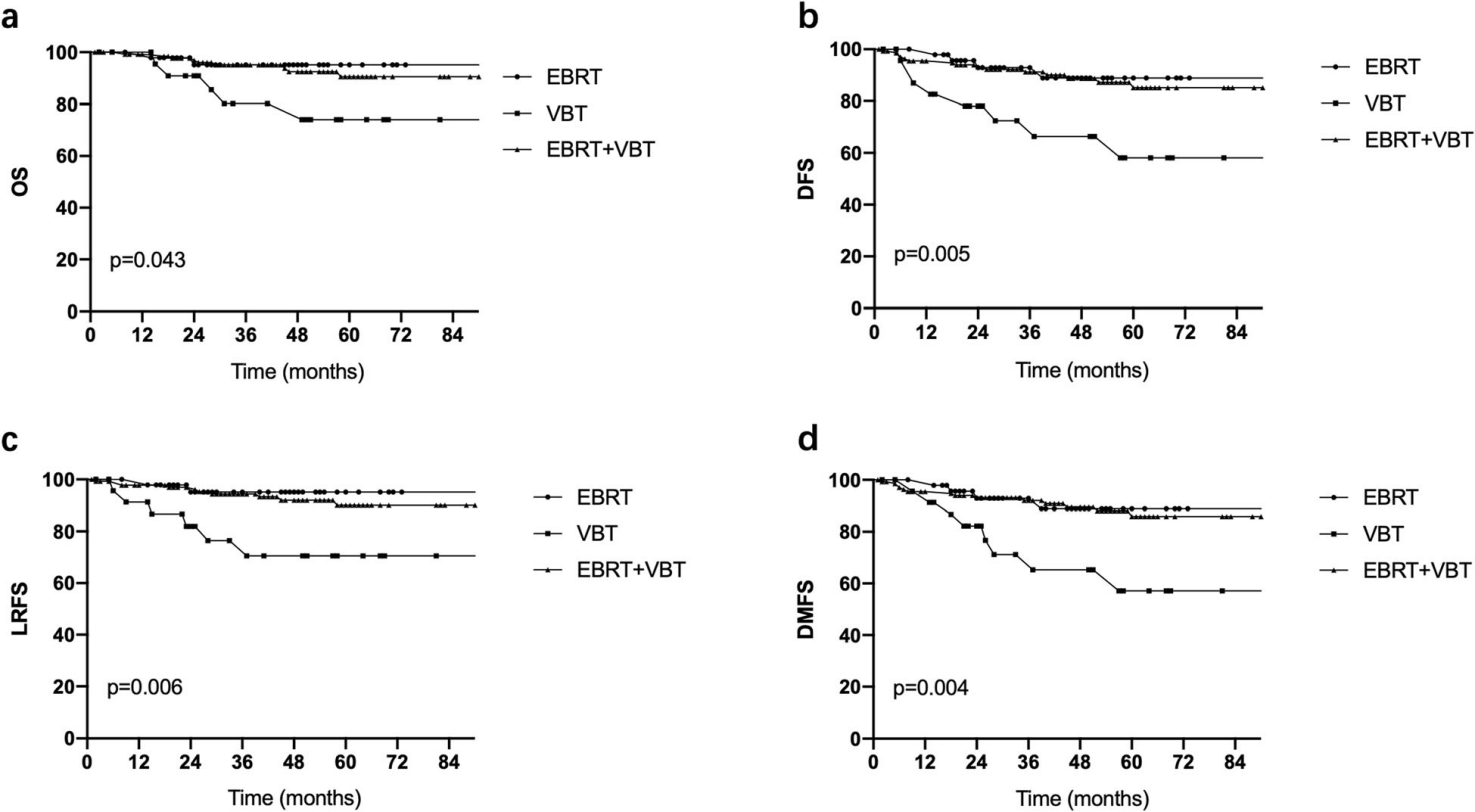
Effect of different adjuvant radiotherapy modalities on survival for patients with stage I to II high-risk endometrioid adenocarcinoma. **a** OS, overall survival; **b** DFS, disease-free survival; **c** LRFS, local-regional failure-free survival; **d** DMFS, distant metastasis failure-free survival

Survival analysis showed that the OS, DFS, LRFS and DMFS were significantly different among the three groups (Fig. 2a, b, c, d). Regarding 3-year OS, the values were 95.2, 85.2 and 95.1% in the EBRT, VBT and EBRT + VBT groups, respectively (*p* = 0.043). The 3-year DFS rates were 92.9 and 72.4% vs. 91.2% for the EBRT and VBT vs. EBRT + VBT groups, respectively (*p* = 0.005). For patients in the VBT group, the 3-year LRFS rate was 76.5%, while the rates were 95.2 and 94.4% in the EBRT group and EBRT + VBT group, respectively (*p* = 0.006).

The groups were compared pairwise with each other, and the results demonstrated that DFS, LRFS and DMFS were worse in the VBT group than in the EBRT (*p* < 0.05) or EBRT + VBT group (*p* < 0.05). OS was significantly improved in the EBRT + VBT group compared to the VBT group. There was no significant difference in survival outcomes between the EBRT group and the EBRT + VBT group.

**Fig. 3:**
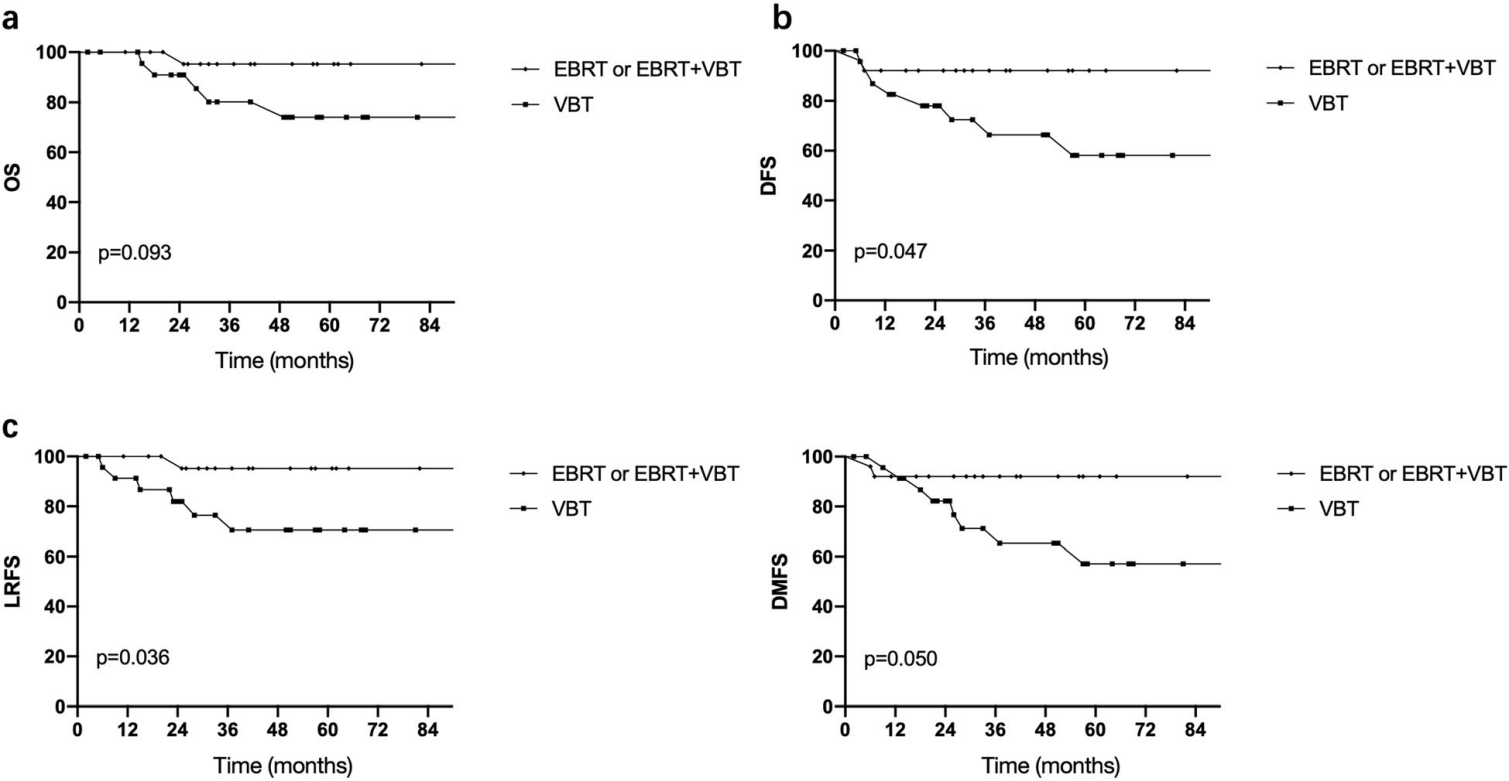
Effect of different adjuvant radiotherapy modalities on survival for patients with stage I to II high-risk endometrioid adenocarcinoma after propensity-matched analysis. **a** OS, overall survival; **b** DFS, disease-free survival; **c** LRFS, local-regional failure-free survival; **d** DMFS, distant metastasis failure-free survival

As the frequency of stage IB and Grade 3 disease were significantly higher and the median age was older in the VBT group than in the EBRT or EBRT + VBT group, A propensity-matched analysis based on stage, grade and age was performed. Fifty patients were successfully matched (25 patients in the VBT group and 25 patients in the EBRT (3 cases) or EBRT + VBT group (21 cases)). Other factors, such as LVSI, deep myometrial invasion and invasion of the lower uterine segment, were equally comparable (*p* > 0.05). The results demonstrated that DFS (*p* = 0.047) and LRFS (*p* = 0.036) were significantly improved in the pelvic radiation group compared to the VBT group (Fig. 3a, b, c, d). In addition, external radiation tended to improve OS and DMFS with no crossing of the survival curves.

### Effects of chemotherapy on survival

For all patients, chemotherapy did not significantly improve survival compared to patients without chemotherapy. Subgroup analysis showed that chemotherapy tended to increase OS, DFS, LRFS and DMFS in patients with LVSI (*n* = 49) and deep myometrial invasion (*n* = 151), and the survival curves did not cross over each other.

### Failure pattern

A total of 25 relapses (11.5%) were found. The median time to recurrence was 18 months (range, 2 months to 60 months). Distant failure was the main failure pattern (12.6%, 23/218). Local and regional recurrences were diagnosed in 4 women (2.8%) in the VBT group and 4 (16%) in the EBRT + VBT group (*p* = 0.008); of these, 1 in the VBT group had isolated local recurrences (*p* = 0.013). Patients in the VBT group had significantly increased local and regional recurrence rates than patients in the EBRT or EBRT + VBT group. However, there was no significant difference on failure between the EBRT and EBRT + VBT groups (Table 2).

**Table 2:** Failure pattern for patients treated with adjuvant EBRT with / or VBT

	Patients (*N* = 218)			
	EBRT (***n*** = 51)	VBT (***n*** = 25)	EBRT + VBT (***n*** = 142)	***p***-value
**Failure mode**	No. (%)	No. (%)	No. (%)	
**Local**	0 (0.0)	2 (8.0)	0 (0.0)	0.013
^a^	^a^ (−0.8)	^a^ (3.9)	^a^ (−1.9)	
**Regional**	0 (0.0)	3 (12.0)	4 (2.8)	0.032
^a^	^a^ (−1.5)	^a^ (2.6)	^a^ (−0.5)	
**Local and Regional**	0 (0.0)	4 (16.0)	4 (2.8)	0.008
^a^	^a^ (−1.6)	^a^ (3.5)	^a^ (−0.9)	
**Distant**	4 (7.8)	6 (24.0)	13 (9.2)	0.065
^a^	^a^ (−0.7)	^a^ (2.3)	^a^ (−0.9)	

*Abbreviation*: *EBRT* External Beam Radiation, *VBT* Vaginal Brachytherapy

^a^ adjusted residuals

### Toxicity

Regarding the toxicity of irradiation, all patients completed the prescription dose of adjuvant radiotherapy. There was no radiation-induced death. Regarding acute adverse events, there was only one case of a grade 4 reaction in the combined radiotherapy group. The most common acute adverse effect was a lower gastrointestinal tract reaction (Grade 1–3, 55.5%). There was no significant difference in radiation-induced cystitis among the three groups. There were significantly lower rate of enterotoxicity and hematological reactions in the VBT group than in the other two groups. Compared with the EBRT group, the rate of gastrointestinal reactions was significantly increased in the EBRT + VBT group (Table 3). Regarding late adverse events, there were no Grade 3 or higher reactions. Lower extremity edema accounted for most among the patients (Grade 1–2, 13.3%). There were no significant differences in radiation-induced enterotoxicity, cystitis or hematological reactions among the three groups.

**Table 3:** Acute adverse effects for patients treated with adjuvant EBRT with/or VBT

	Patients (*N* = 218)			
	EBRT (***n*** = 51)	VBT (***n*** = 25)	EBRT + VBT (***n*** = 142)	***p***-value
**Adverse Events**	No. (%)	No. (%)	No. (%)	
^**b**^**Upper GI**				0.001
0	36 (70.6)	21 (84.0) ^a^2.5	77 (54.2) ^a^-3.0	
1–2	15 (29.4)	3 (12.0) ^a^-2.9	65 (45.8) ^a^3.2	
3	0 (0.0)	1 (4.0) ^a^2.8	0 (0.0)	
^**b**^**Lower GI**				0.000
0	27 (52.9)	22 (88.0) ^a^4.7	48 (33.8) ^a^-4.3	
1–2	23 (45.1)	3 (12.0) ^a^-4.5	93 (65.5) ^a^4.4	
3	1 (2.0)	0 (0.0)	1 (0.7)	
**Urinary Tract**				0.864
0	44 (86.3)	23 (92.0)	123 (86.6)	
1–2	7 (13.7)	2 (8.0)	18 (12.7)	
3	0 (0.0)	0 (0.0)	1 (0.7)	
**Hematological**				0.023
0	23 (45.1)	21 (84.0) ^a^3.1	75 (52.8)	
1–2	25 (49.0)	4 (16.0) ^a^-2.7	60 (42.3)	
3–4	3 (5.9)	0 (0.0)	7 (4.9)	

*Abbreviation*: *GI* gastrointestinal, *EBRT* External Beam Radiation, *VBT* Vaginal Brachytherapy

^a^ adjusted residuals, only value greater than ±2 were marked. ^b^ Upper GI toxicity defined in this study included nausea and vomiting; Lower GI reactions included diarrhea, constipation, abdominal pain et al

## Discussion

For stage I to II high-risk endometrioid adenocarcinoma, we reported that compared with VBT alone, adjuvant pelvic external radiation, including EBRT or EBRT combined with VBT, significantly improved the DFS, LRFS and DMFS with good compliance and safety. Overall survival was significantly prolonged in the EBRT + VBT group. There was no significant difference in survival outcomes between the EBRT and EBRT + VBT modalities.

The indication and modality of adjuvant radiotherapy is risk-based. For patients with intermediate or high-intermediate risk factors, prospective trials comparing postoperative EBRT to no adjuvant therapy showed that postoperative radiotherapy increased the local-regional control rate [4–6]. However, it did not translate into survival benefits. The PORTEC-2 trial [7, 8] directly compared EBRT with vaginal brachytherapy and concluded that VBT was no less effective than EBRT in terms of locoregional control rate and survival outcome. Aalders’ randomized trial [9] found that, compared to VBT, combined EBRT and VBT reduced locoregional recurrences but did not reduce distant metastases or improve survival. Vaginal brachytherapy alone is currently recommended for intermediate- or high-intermediate-risk cases (FIGO stage IA and Grade 3 disease or FIGO stage IB and Grade 1/2 disease).

The use of vaginal brachytherapy alone in the high-risk subset is still under debate. Prospective trials, such as the PORTEC-1 and PORTEC-2 trials, specifically excluded patients with stage IB (FIGO 2009) Grade 3 endometrial cancer, while other studies only included a small number of higher-risk patients. Data from SEER analyses found that among patients with FIGO stage IB and Grade 3 disease who underwent lymph node dissection, all three radiotherapy modalities (EBRT, EBRT + VBT and VBT alone) were superior to no adjuvant treatment, without significant differences among the radiation modalities [10]. Another research from SEER database concluded VBT (with or without EBRT) might confer a cancer-specific survival benefit in stage IB Grade 3 patients. Regional treatment with EBRT and lymph node dissection were critically important in high-grade stage II disease [11]. However, a 2018 published research from SEER database demonstrated there was no difference in disease specific survival when VBT alone was compared with EBRT alone or both for Grade 2–3 stage II disease [12].

In this research, we explored the optimal adjuvant radiotherapy mode for patients with high-risk endometrioid adenocarcinoma after total hysterectomy and bilateral salpingo-oophorectomy and mostly lymphadenectomy (87.2%). In terms of survival, DFS, LRFS and DMFS were significantly higher in patients treated with pelvic external radiation than in those treated with VBT alone. Overall survival was significantly improved in the EBRT + VBT group. Previous studies have shown that, compared with VBT alone, adjuvant external irradiation could reduce the pelvic recurrence rate [13] while controlling vaginal stump relapse. However, in patients with low- or intermediate-risk factors, the locoregional recurrence rate was low due to the low degree of malignancy. Other trials demonstrated that the 5-year locoregional recurrence rate was 6.1% in the ASTEC observation group [4], 6.9% in the Aalder VBT group [9] and 5.1% in the PORTEC2 VBT group [8]. Therefore, substitution of VBT with EBRT + VBT or EBRT could not significantly reduce the locoregional recurrence rate; even if it was reduced, it could not translate into survival benefits [8, 9, 13]. With the upgrade of stage and differentiation, the failure rate of local, regional and distant areas increased. For patients receiving 46 Gy of pelvic radiation in the PORTECT1 trial [14], the actuarial 5-year rates of locoregional relapse were 1 to 3% for IC, Grade 1–2, compared with 14% for stage IC, Grade 3 patients. The five-year distant metastasis rates were 20% for stage IB and Grade 3 tumors and 31% for stage IC and Grade 3 tumors (FIGO 1988). Due to a high degree of malignancy, high-risk patients had high local, pelvic and distant failure rates. Therefore, external irradiation could effectively reduce pelvic and vaginal failure rates and indirectly play a role in reducing distant recurrence, ultimately benefiting survival. In our research, patients in VBT group had older age, higher grade, which were known as potential risk factors on survival and might influence the outcome. A propensity-matched analysis based on stage, grade and age was performed, and results still showed an improved survival towards EBRT.

In this study, we reported that survival was not significantly different for combined EBRT and VBT compared to EBRT alone. VBT was effective in vaginal stump recurrence control, while pelvic radiation offered the possibility of treating the vaginal area in addition to the pelvic lymphatic drainage area. Among deeply myometrial invasive Grade 3 patients (stage IB, G3) after pelvic radiation, the vaginal recurrence rate was only 5% [14]. Among cervical involvement patients (stage II), there was no suggestion that the addition of a vaginal cuff brachytherapy boost to pelvic radiation was beneficial for pelvic control (84% vs. 95%, *p* = 0.43) or survival [15]. Analysis from SEER demonstrated a non-significant survival difference between combined EBRT and VBT or EBRT alone in stage IB, G3 or stage II patients [11]. Therefore, for high-risk patients, the local-regional control rate of EBRT alone was acceptable. A VBT boost seemed to be unable to improve the pelvic and vaginal control rate or overall survival. However, whether external irradiation should be combined with internal irradiation is still under debate.

Regarding failure mode, the results showed that the locoregional recurrence rate of patients with pelvic irradiation, was significantly lower and that the distant failure rate tended to be lower than that of VBT alone, which emphasized the importance of EBRT in high-risk populations. In addition, distant metastasis was the main cause of failure in this study, which was similar to other studies [16, 17]. Chemotherapy might eliminate systemic micro-metastases, especially distant metastases and benefit survival. Currently, studies had been conducted to evaluate the efficacy of chemotherapy for patients with high-risk endometrial cancer by replacing pelvic radiation or using it as sequential or concurrent adjuvant therapy [18–21]. The PORTECT3 trial [22] showed for early stage high-risk patients (stage IB patients with Grade 3 or LVSI and stage II patients), chemoradiotherapy didn’t significantly improve 5-year failure-free survival compared to radiotherapy alone. GOG-0249 [23] was designed to study the impact on recurrence-free survival of substitution of VBT with chemotherapy for EBRT among patients with high-risk stage I to II endometrial cancer. However, superiority of VBT with chemotherapy compared to EBRT was not demonstrated. In our research, chemotherapy did not significantly improve survival. Anyway, risk factors such as LVSI and deep myometrial invasion still warranted attention.

In terms of toxicity, acute toxicity was well tolerated in all patients, with only one patient representing a Grade 4 acute response. No adverse reactions of Grade 3 or above were found in the follow-up period after treatment. Currently, many studies are combining molecular targeted therapy with clinicopathological risk factors, which can distinguish patients who require less or more intensified adjuvant treatment [24, 25]. However, more evidence is needed, and randomized trials are necessary.

Some of the limitations were as follows. As a retrospective study, some details about pathology and chemotherapy were not complete. As a muti-center study, clinical and pathology records and treatment standards might not be unified across institutions. Statistically speaking, the number of patients in the three groups were not equally distribute, which might affect the efficiency of statistical calculation. However, this was the largest retrospective multicenter research focusing on selection of EBRT with/or VBT in stage I-II high risk endometrioid adenocarcinoma patients.

## Conclusions

For patients with FIGO (2009) stage I to II endometrioid adenocarcinoma, adjuvant radiotherapy including pelvic external radiation significantly improved DFS, LRFS and DMFS with good compliance and safety. Survival outcomes were not significantly different between the EBRT and EBRT + VBT modalities. Further validation of these findings is necessary in the future.

## Data Availability

The datasets used and analyzed during the current study are available from the corresponding author upon reasonable request.

## Abbreviations

EC: Endometrial cancer
EBRT: External beam radiation
VBT: Vaginal brachytherapy
ESMO-ESGO-ESTRO: European Society for Medical Oncology, European Society for Radiotherapy & Oncology and European Society of Gynaecological Oncology
OS: Overall survival
DFS: Disease free survival
LRFS: Local-regional failure free survival
DMFS: Distant metastasis failure free survival
ANOVA: Analysis of variance
PSM: Propensity-matched analysis
IMRT: Intensity modulated radiation therapy
CTV: Clinical target volume
PTV: Planning target volume
HDR: High-dose rate
3D-CRT: Three-dimensional conformal radiotherapy
FIGO: International Federation of Gynecology and Obstetrics
CTCAE 4.0: Common Terminology Criteria for Adverse Events Version 4.0
SEER: Surveillance, Epidemiology, and End Results Database
TH+ BSO: Total hysterectomy and bilateral salpingo-oophorectomy
SEER: Surveillance, Epidemiology, and End Results

## References

[1] Chen W, Zheng R, Baade PD, Zhang S, Zeng H, Bray F, Jemal A, Yu XQ, He J (2016). Cancer statistics in China, 2015. CA Cancer J Clin.

[2] Colombo N, Creutzberg C, Amant F, Bosse T, González-Martín A, Ledermann J, Marth C, Nout R, Querleu D, Mirza MR, Sessa C, Abal M, Altundag O, Amant F, van Leeuwenhoek A, Banerjee S, Bosse T, Casado A, de Agustín LC, Cibula D, Colombo N, Creutzberg C, del Campo JM, Emons G, Goffin F, González-Martín A, Greggi S, Haie-Meder C, Katsaros D, Kesic V, Kurzeder C, Lax S, Lécuru F, Ledermann J, Levy T, Lorusso D, Mäenpää J, Marth C, Matias-Guiu X, Morice P, Nijman HW, Nout R, Powell M, Querleu D, Mirza MR, Reed N, Rodolakis A, Salvesen H, Sehouli J, Sessa C, Taylor A, Westermann A, Zeimet AG (2016). ESMO-ESGO-ESTRO consensus conference on endometrial Cancer: diagnosis, treatment and follow-up. Ann Oncol.

[3] Amant F, Moerman P, Neven P, Timmerman D, van Limbergen E, Vergote I (2005). Endometrial cancer. Lancet.

[4] Blake P (2009). Adjuvant external beam radiotherapy in the treatment of endometrial cancer (MRC ASTEC and NCIC CTG EN.5 randomised trials): pooled trial results, systematic review, and meta-analysis. Lancet.

[5] Scholten AN, van Putten WLJ, Beerman H, Smit VTHBM, Koper PCM, Lybeert MLM, Jobsen JJ, Wárlám-Rodenhuis CC, de Winter KAJ, Lutgens LCHW, van Lent M, Creutzberg CL (2005). Postoperative radiotherapy for stage 1 endometrial carcinoma: long-term outcome of the randomized PORTEC trial with central pathology review. Int J Radiat Oncol Biol Phys.

[6] Keys HM, Roberts JA, Brunetto VL, Zaino RJ, Spirtos NM, Bloss JD, Pearlman A, Maiman MA, Bell JG, Gynecologic Oncology Group (2004). A phase III trial of surgery with or without adjunctive external pelvic radiation therapy in intermediate risk endometrial adenocarcinoma: a gynecologic oncology group study. Gynecol Oncol.

[7] Wortman BG (2018). Ten-year results of the PORTEC-2 trial for high-intermediate risk endometrial carcinoma: improving patient selection for adjuvant therapy. Br J Cancer.

[8] Nout RA, Smit VTHBM, Putter H, Jürgenliemk-Schulz IM, Jobsen JJ, Lutgens LCHW, van der Steen-Banasik E, Mens JWM, Slot A, Kroese MCS, van Bunningen B, Ansink AC, van Putten W, Creutzberg CL (2010). Vaginal brachytherapy versus pelvic external beam radiotherapy for patients with endometrial cancer of high-intermediate risk (PORTEC-2): an open-label, non-inferiority, randomised trial. Lancet.

[9] Aalders J, Abeler V, Kolstad P, Onsrud M (1980). Postoperative external irradiation and prognostic parameters in stage I endometrial carcinoma: clinical and histopathologic study of 540 patients. Obstet Gynecol.

[10] Chino JP, Jones E, Berchuck A, Secord AA, Havrilesky LJ (2012). The influence of radiation modality and lymph node dissection on survival in early-stage endometrial cancer. Int J Radiat Oncol Biol Phys.

[11] Xiang M, Kidd EA (2020). Survival benefit of radiation in high-risk, early-stage endometrioid carcinoma. J Gynecol Oncol.

[12] Nwachukwu CR, Von-Eyben R, Kidd EA (2018). Radiation therapy improves disease-specific survival in women with stage II endometrioid endometrial cancer-brachytherapy may be sufficient. Brachytherapy.

[13] Sorbe B, Horvath G, Andersson H, Boman K, Lundgren C, Pettersson B (2012). External pelvic and vaginal irradiation versus vaginal irradiation alone as postoperative therapy in medium-risk endometrial carcinoma--a prospective randomized study. Int J Radiat Oncol Biol Phys.

[14] Creutzberg CL, van Putten W, Wárlám-Rodenhuis CC, van den Bergh A, de Winter KA, Koper PC, Lybeert ML, Slot A, Lutgens LC, Stenfert Kroese MC, Beerman H, van Lent M, postoperative Radiation Therapy in Endometrial Carcinoma Trial (2004). Outcome of high-risk stage IC, grade 3, compared with stage I endometrial carcinoma patients: the postoperative radiation therapy in endometrial carcinoma trial. J Clin Oncol.

[15] Greven KM, D’Agostino RB, Lanciano RM, Corn BW (1998). Is there a role for a brachytherapy vaginal cuff boost in the adjuvant management of patients with uterine-confined endometrial cancer?. Int J Radiat Oncol Biol Phys.

[16] Jung J, Kim YS, Joo JH, Park W, Lee JH, Kim JH, Yoon WS, Lee SH, Eom KY, Kim YB (2017). Oncologic outcomes after adjuvant radiotherapy for stage II endometrial carcinoma: a Korean radiation oncology group study (KROG 14-10). Int J Gynecol Cancer.

[17] Setakornnukul J, Petsuksiri J, Wanglikitkoon S, Warnnissorn M, Thephamongkhol K, Chansilp Y, Veerasarn V (2014). Long term outcomes of patients with endometrial carcinoma treated with radiation - Siriraj hospital experience. Asian Pac J Cancer Prev.

[18] Elemam O (2020). Sequential chemoradiotherapy compared to radiotherapy in endometrial carcinoma. Asian Pac J Cancer Prev.

[19] Laliscia C, Cosio S, Morganti R, Mazzotti V, Fabrini MG, Paiar F, Gadducci A (2019). Patterns of failures and clinical outcome of patients with early-stage, high-risk, node-negative endometrial Cancer treated with surgery followed by adjuvant platinum-based chemotherapy and vaginal brachytherapy. Oncology.

[20] Smogeli E, Cvancarova M, Wang Y, Davidson B, Kristensen G, Lindemann K (2018). Clinical outcome of patients with high-risk endometrial carcinoma after treatment with chemotherapy only. Int J Gynecol Cancer.

[21] Reynaers EA, Jutzi L, Ezendam NPM, Kwon JS, Pijnenborg JMA (2017). Improved outcome of high-grade, early 1-stage Endometrioid endometrial carcinoma with adjuvant chemotherapy and radiotherapy: comparison of 2 treatment strategies. Int J Gynecol Cancer.

[22] de Boer SM, Powell ME, Mileshkin L, Katsaros D, Bessette P, Haie-Meder C, Ottevanger PB, Ledermann JA, Khaw P, Colombo A, Fyles A, Baron MH, Jürgenliemk-Schulz IM, Kitchener HC, Nijman HW, Wilson G, Brooks S, Carinelli S, Provencher D, Hanzen C, Lutgens LCHW, Smit VTHBM, Singh N, Do V, D'Amico R, Nout RA, Feeney A, Verhoeven-Adema KW, Putter H, Creutzberg CL, McCormack M, Whitmarsh K, Allerton R, Gregory D, Symonds P, Hoskin PJ, Adusumalli M, Anand A, Wade R, Stewart A, Taylor W, Kruitwagen RFPM, Hollema H, Pras E, Snyers A, Stalpers L, Jobsen JJ, Slot A, Mens JWM, Stam TC, van Triest B, van der Steen - Banasik EM, de Winter KAJ, Quinn MA, Kolodziej I, Pyman J, Johnson C, Capp A, Fossati R, Gribaudo S, Lissoni AA, Ferrero A, Artioli G, Davidson C, McLachlin CM, Ghatage P, Rittenberg PVC, Souhami L, Thomas G, Duvillard P, Berton-Rigaud D, Tubiana-Mathieu N (2018). Adjuvant chemoradiotherapy versus radiotherapy alone for women with high-risk endometrial cancer (PORTEC-3): final results of an international, open-label, multicentre, randomised, phase 3 trial. Lancet Oncol.

[23] Randall ME, Filiaci V, McMeekin DS, von Gruenigen V, Huang H, Yashar CM, Mannel RS, Kim JW, Salani R, DiSilvestro PA, Burke JJ, Rutherford T, Spirtos NM, Terada K, Anderson PR, Brewster WR, Small W, Aghajanian CA, Miller DS (2019). Phase III trial: adjuvant pelvic radiation therapy versus vaginal brachytherapy plus paclitaxel/carboplatin in high-intermediate and high-risk early stage endometrial Cancer. J Clin Oncol.

[24] Wortman BG, Bosse T, Nout RA, Lutgens LCHW, van der Steen-Banasik E, Westerveld H, van den Berg H, Slot A, de Winter KAJ, Verhoeven-Adema KW, Smit VTHBM, Creutzberg CL, PORTEC Study Group (2018). Molecular-integrated risk profile to determine adjuvant radiotherapy in endometrial cancer: evaluation of the pilot phase of the PORTEC-4a trial. Gynecol Oncol.

[25] Stubert J, Gerber B (2016). Current issues in the diagnosis and treatment of endometrial carcinoma. Geburtshilfe Frauenheilkd.

